# 

**Published:** 1892-09

**Authors:** 


### LITERARY.

THE PSYCHICAL REVIEW. A Quarterly Journal of Psychical Science and Organ of the American Psychical Society.

Vol. I, No. 1, of this interesting quarterly, is before us, and most heartily do we welcome it as a new recruit in making clear the way of TRUTH.

We confess, that when this Society was first organized, we had little faith in its accomplishment of any great good in the fields to which its enquiries were to be directed, but certainly not from any lack of courage or ability on the part of its projectors, for they, at least some of them, had already made their mark as fearless investigators and outspoken advocates of newly found truths, regardless of their popular estimate.

Perhaps we held too freshly in mind the utter worthlessness of the Siebert Commission, whose "Grave and Reverend Seigniors" failed to find any thing in the whole range of occultism worthy of mention.

We were under the impression too, that the membership of the new-born Society was to be composed mainly of clergymen, concerning whom, as a class, our expectations were not at all buoyant.

The Review in question is a complete negation to our misgivings and a vindication that the objects of the Society as expressed in its constitution, will, so far as possible, be faithfully carried out, viz : "To institute an investigation of the phenomena of Modern Spiritualism in accordance with the scientific method."

Aside from the impracticability, of investigating the phenomena of Modern Spiritualism by ordinary scientific methods, the objects are well stated, but even in this field the Society is far from being in the advance. At best it is merely a gleaner over fields where an ample store has been faithfully harvested. The labors of such men as Professors Hare, Britan, Denton, Kiddle, Buchanan and others of lesser renown are worthy of such a following as the catalogue of membership in this Society shows, and all well meaning spiritualists will welcome their organized efforts to still further fathom the mysteries of which we as individuals form no inconsiderable share.

The frontispiece of the Review is a likeness of the Rev. Minot J. Savage, the author of its leading article ; there is also an able article by Rev. T. E. Allen, its Secretary, with whom the writer in other days, has held frequent converse and searchings concerning the occult.

THE PHYSICIAN HIMSELF ; F. A. Davis & Co., Publisher, Philadelphia.

Some time ago one of our leading Dentists entered upon the publication of a monthly, entitled "The Dentist Himself." We thought it a queer sort of a title, but the book before us, which has climbed to its 10th edition, must have preceded him by long odds.

"Know then thyself," says a famous English poet, but it is advice too frequently lost upon the common mind, for most people are more interested in knowing their neighbors.

We scanned the pages of this volume with much curiosity, and truth to say, were we a physician, we should rather give up the vulgar practice of eating than be without it. The fact is, no physician of any school can half know himself, without this 4to of some 400 pages to refer to when in doubt. The index occupies nearly six pages, from which we infer that the true physician must be a very complex individual, and would not have much leisure time to experiment upon the sick if he studied himself thoroughly, which he certainly ought to do, with this book in hand.

REPORT ON ABDOMINAL AND PELVIC SURGERY, including 32 Successful Cases of Laparotomy ; by Professor William H. Wathen, Louisville, Ky. A very valuable pamphlet.

